# Common European Origin of Hepatitis E Virus in Human Population From Eastern Romania

**DOI:** 10.3389/fpubh.2020.578163

**Published:** 2020-12-17

**Authors:** Daniela Porea, Adriana Anita, Andrei Vata, Danut Teodor, Luciana Crivei, Cristian Raileanu, Vasilica Gotu, Ioana Ratoi, Andreea Cozma, Dragos Anita, Luanda Oslobanu, Nicole Pavio, Gheorghe Savuta

**Affiliations:** ^1^Department of Public Health, Faculty of Veterinary Medicine, University of Agricultural Sciences and Veterinary Medicine of Iasi, Iaşi, Romania; ^2^Center for the Study of Transborder and Emergent Diseases and Zoonoses Department, Danube Delta National Institute for Research and Development, Tulcea, Romania; ^3^“Sfanta Parascheva” Infectious Diseases Hospital of Iasi, Iaşi, Romania; ^4^Laboratory of Vector Capacity, Institute of Infectology, Friedrich-Loeffler-Institut, Greifswald, Germany; ^5^Department of Parasitology and Parasitic Diseases and Animal Biology, Faculty of Veterinary Medicine, University of Agronomical Sciences and Veterinary Medicine of Bucharest, Bucharest, Romania; ^6^UMR Virologie 1161, ENVA, INRAE, Anses, Maisons-Alfort, France

**Keywords:** hepatitis E virus, human population, acute hepatitis, zoonotic character, phylogenetic analysis

## Abstract

The purpose of this research was to improve the epidemiological data on HEV infection in the human population in Romania. The analysis targeted hospitalized subjects with acute hepatitis (*n* = 94) of unknown etiology from the Infectious Diseases Regional Hospital in Iasi. Moreover, patients without liver disease (*n* = 40) from a different county hospital located in Eastern Romania were included. The presence of HEV infection and first characterization of human HEV strains was determined using serological and molecular assays. The apparent HEV seroprevalence varied between 29.16% (95% CI, 16.31–42.03) and 32.5% (95% CI, 17.98–47.02) according to patient grouping. Molecular analysis enhanced the detection of two HEV isolates, that clustered in subtype HEV-3c, the most commonly identified subtype in Europe. Identification of acute hepatitis E cases, together with the first detection and molecular characterization of human HEV in Romania represent the originality attributes of the present study.

## Introduction

Hepatitis E virus (HEV) infection is an emerging *health* concern worldwide. HEV can be transmitted from human to human via the fecal-oral route or from animals to humans through feces, direct contact, or consumption of contaminated meat products. HEV is a quasi-enveloped RNA virus with a single-stranded, positive-sense genome, classified in the *Hepeviridae* family (1, 2). HEV infections are common in humans and animals, being distributed globally in both developing and industrialized countries. Hepatitis E virus strains that infects humans belongs to the *Orthohepevirus* A species. Genotypes 1 and 2 (HEV-1 and HEV-2) only infect humans and are endemic to Asia, Africa, and Central America, where they cause large, usually waterborne, hepatitis epidemics, whereas zoonotic genotypes 3 and 4 (HEV-3 and HEV-4) cause sporadic cases worldwide (3). In European countries, the incidence of confirmed HEV cases has been steadily increasing over the last decade, highlighting the relevance and emerging nature of this zoonotic infection (4). HEV-3 predominates in high-income countries, including those in Europe. In these countries HEV causes an acute infection, which may be associated with clinical hepatitis and can also result in a persistent infection in immunosuppressed hosts. Hepatitis E virus is also an under-recognized cause of neurological conditions, including brachial neuritis and peripheral neuropathy (5). The infections with HEV-4 have been described in France, Italy and Germany (6). Our understanding of HEV infection has changed radically in the last decade, with HEV being currently considered a global threat to human health. The zoonotic character of HEV infection was highlighted by direct and indirect evidence, the latter being preponderant. Indirect evidences, based on the genetic association between human and animal isolates, point out that not all animal HEV strains may be associated with zoonotic infection (7). Of the susceptible animals to HEV infection, domestic pig and wild boars are the main sources of the virus. In Europe, the consumption of raw or undercooked meat from infected animals appears to be the major transmission route for HEV infection in humans (8). Since 2010, hepatitis E infection has been detected in Romania, in swine (9) and wild boars (10). In parallel there are serological evidences of HEV infections in human (11, 12). The epidemiology of hepatitis E infections in the Romanian population remains poorly described, as HEV infection is not a notifiable disease. HEV infections are probably underdiagnosed, as are either not reported or go unnoticed, in cases of mild or asymptomatic clinical forms. The main purpose of this pilot study was to investigate the current seroprevalence of anti-HEV antibodies in different population groups and to identify HEV genotype and subtypes circulating in human population in Romania. To provide epidemiological and etiological data on HEV infection, serological, molecular methods and bioinformatics analysis were undertaken.

## Materials and Methods

### Study Population

A longitudinal study was conducted between 2016 and 2017. For HEV detection in humans, biological specimens (serum, plasma or feces) were sampled from three locations in Eastern Romania: “Sfanta Parascheva” Infectious Diseases Hospital of Iasi (SPIDHI), Public Health Authority (PHA) from Iaṣi and Vrancea County. Samples provided by Public Health Authorities originated from patients hospitalized in SPIDHI and Emergency County Hospital “St. Pantelimon,” Focṣani (ECHSPF).

The samples from SPIDHI, were collected after obtaining the written informed consent from all patients or the parent/legal guardian in case of <18 years of age participants, as appropriate. Approval for acquiring the samples was based on the Decision No. 8/18.02.2015, issued by the Ethics Commission of the “Sfanta Parascheva” Infectious Diseases Hospital of Iasi. The Ethics Commission, approved the request made by the University Center for Veterinary Medical Research from University of Agricultural Sciences and Veterinary Medicine of Iasi, to carry out the necessary research for the PhD thesis entitled: “Epidemiological and Etiological Investigations on Hepatitis E Virus Infection in Wild Animals and Assessment of Zoonotic Risk” authored by Daniela Porea.

Following the collaboration with PHA Iasi and PHA Vrancea, we included in this study human sera samples, tested for various infections within the serology laboratories of these institutions.

### Sampling and Storage

The samples were divided into two categories: (1) from patients with acute hepatitis and (2) from patients without liver disease.

The samples collected from SPIDHI originated from patients (*n* = 48) with acute hepatitis of unknown etiology (patients with clinical symptoms of hepatitis in which the most frequent causes were excluded: hepatic viruses, alcohol and drug use, as well as the uncommon causes: other viral infections and leptospirosis). Out of these, only one originated from a person who had traveled abroad during the sampling year. The collected data revealed that the patients included in the study are pork meat consumers. The patients were divided according to age category and the sampling unit.

The study also included serum samples (*n* = 16) from the hospital's medical staff, aged between 27 and 46 years.

The samples from the PHA Iasi (*n* = 46) were obtained from the batch of samples serological previously in the institution's own laboratory and identified as negative for viral hepatitis A (HAV) and B (HBV) infections (non-A, non-B), between May 2016 and March 2017, most of them during 2016.

**Figure 1:**
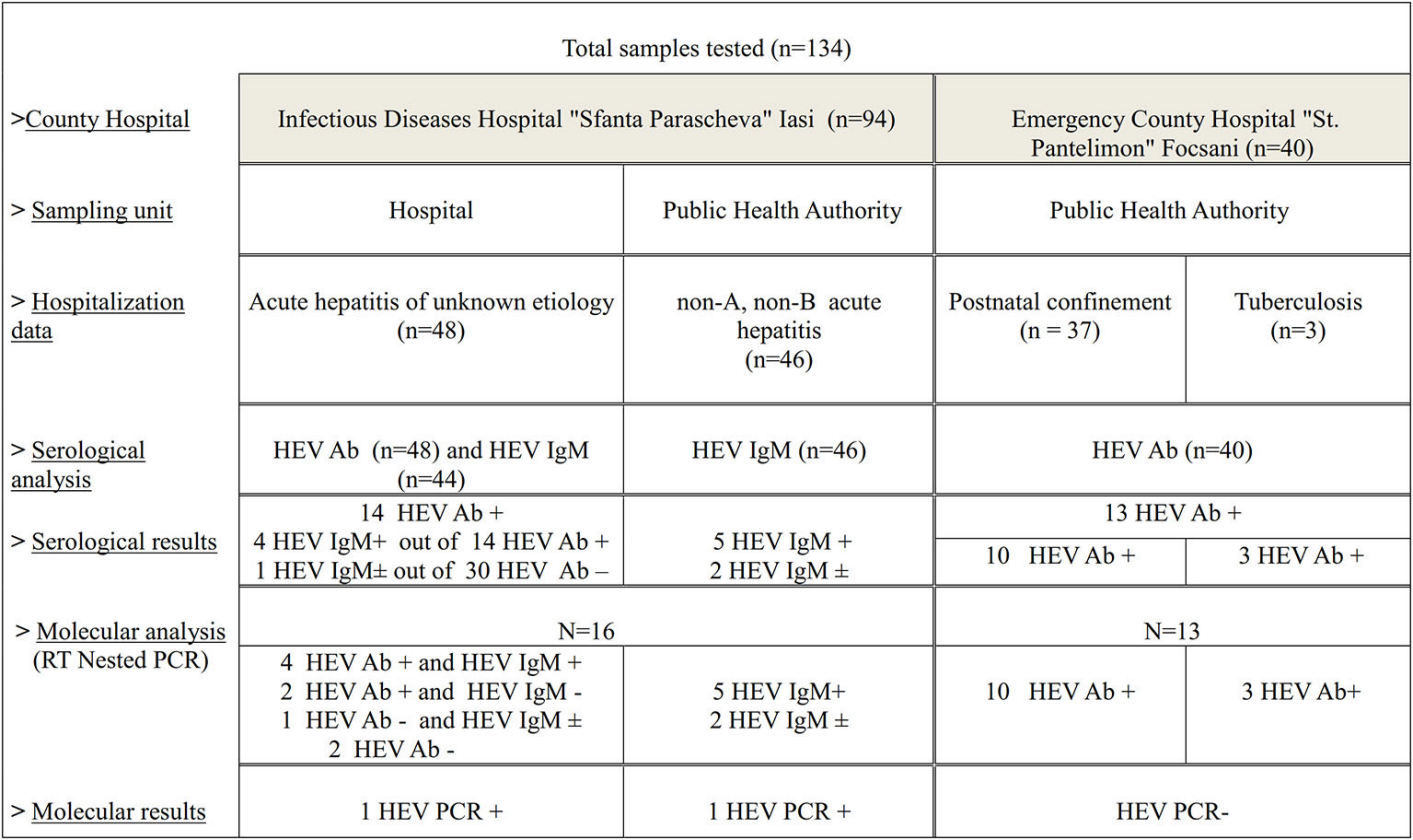
Diagram of the study flow chart.

From PHA Vrancea, the obtained samples (*n* = 40) were tested negative for HIV infection during the surveillance National Program of Prevention, Surveillance and Control of HIV/AIDS (I.3), which monitors the vertical transmission of the HIV virus, from the mother to the fetus, and the risk of HIV presence in patients with tuberculosis. In 2017, from the same county, samples were collected from hospitalized patients in ECHSPF: obstetrics-gynecology section (postnatal confinement, *n* = 37) and Tuberculosis section (*n* = 3) (Figure 1). These patients have not presented characteristic symptoms of liver disease. The patients are originated from both rural and urban area in Vrancea County. Samples were stored as following: serum samples at −20°C and plasma and feces samples at −80°C until use.

### Serological Analysis

ELISA HEV total Ab and ELISA HEV IgM (Diagnostic Bioprobes, Italy) kits were used in order to identify anti-HEV antibodies in human serum samples. The protocol used for each analysis was performed according to the manufacturer′s instruction. Serum samples from the SPIDHI patients were initially tested for the presence of total antibodies against hepatitis E virus (Ab) and subsequently the positive samples and some of the negative ones (randomly selected) were retested for the identification of IgM anti-HEV antibodies, together with those from PHA Iasi (Figure 1). The samples collected from medical staff and those obtained from PHA Vrancea were tested for the presence of HEV total Ab. Test results were interpreted as ratio of the sample OD450 nm and the Cut-Off value (or S/Co) according to the following values: S/Co <0.9 for negative results, between 0.9 and 1.1 for undetermined, S/Co > 1.1 for positive results.

### Gene Amplification - RT-Nested PCR

All anti-HEV Ab positive sera and samples (serum, plasma or feces samples depending on their availability) from hospitalized patients were analyzed for molecular detection of HEV RNA. RNA extraction was performed using the QIAamp Viral RNA Mini Kit (Qiagen, reference 52904, Hilden, Germany), according to the manufacturer's instructions. For feces samples, the protocol underwent adjustments according to Anita et al. (9). For HEV RNA detection, the RT-nested PCR method was applied, using two sets of degenerate primers (13). The assay was adapted from the method described by Cooper et al. (13) and performed as described by Bouquet et al. (14) by using the FIREPol® DNA Polymerase reaction kit (Tartu, Estonia) and the dNTP Mix kit (Promega, Madison, USA). The nested PCR products were analyzed after migration on agarose gel (1%) and ethidium bromide staining. The expected final product of the nested RT-PCR was 348 bp.

### Nucleotide Sequencing and Phylogenetic Analysis

Sequencing of positive amplicons was performed by BASECLEAR (Netherlands) using the Sanger's method. Sequencing of PCR products was performed for each strand using the inner primers: 3158 and 3159. Nucleotide sequences were analyzed and individually edited using Bioedit software. The sequences were loaded to the alignment explorer in MEGA 10 (15) and aligned with ClustalW (16). Datasets added for alignment included also the reference sequences (17). A model test was run in MEGA X prior to the construction of the tree for the selection of the suitable model. The phylogenetic tree was built using the Maximum Likelihood method based on the Kimura 2-parameter model (18) with 1,000 replicates. A human HEV-1 strain (EF530670.1/) was used as out group.

### Statistical Analyses

The statistical analysis was performed using the Social Science Statistics website (http://www.socscistatistics.com), using the Fisher Exact Test. *p*-values lower than 0.10 were considered significant. The 95% confidence interval (CI) calculation was performed using MS Excel 2016, predefining the calculation formula.

## Results

### Serological Results

All the samples collected from the hospital staff (*n* = 16) were identified as Ab HEV negative following the serological analysis.

Total HEV antibodies were detected in 14 out of 48 samples analyzed from patients with acute hepatitis of unknown etiology, with an overall prevalence of 29.16% (95% CI, 16.31–42.03) The reported seroprevalence ranged according to the age group (Table 1), with significant differences observed between the first two categories and the last category taken as reference (*p* = 0.06 and *p* = 0.08, respectively). Anti-HEV IgM antibodies were detected in 4 out of the 14 positive Ab HEV samples. For one HEV Ab negative serum (randomly selected), an ambiguous HEV IgM result (S/Co = 1.19) was obtained.

**Table 1:** Seroprevalence of total anti-HEV antibodies in patients with acute hepatitis of unknown etiology.

**Age category**	**Seroprevalence**	**Confidence interval (95%)**	***P*-value**
1–20 years	12.5% (1/8)	−10.42–35.42	0.06^*^
21–40 years	23.5% (4/17)	3.37–43.69	0.08^*^
41–60 years	26.7% (4/15)	4.29–49.05	0.1
>60 years	62.5% (5/8)	28.95–96.05	–
Total	29.2% (14/48)	16.31–42.03	

* *P-value < 0.10*.

In case of patients with non-A non-B acute hepatitis the serological exam consisted only in HEV-IgM detection considering the clinical form of the infection. After testing the 46 serums, 5 samples were identified as HEV IgM positive (10.86%, CI, 1.87–19.86), and for two samples were obtained ambiguous results (S/Co = 1.19 and S/Co = 1.17, respectively).

Therefore, 9 out of 90 patients with acute hepatitis serologically tested were IgM anti-HEV positive. For two out of nine HEV IgM positive samples, the cut-off value of the optical density recorded values more than 10 times (S/Co = 12.6 and 13.4, respectively) greater than the minimum reference value (S/Co = 1.2).

Serological screening of HEV Ab in patients without liver disease from PHA Vrancea revealed 13 positive sera (32.5%, CI, 17.98–47.02), of which three positive samples in TB patients and 10 positive samples from women patients in postnatal confinement (27.02%, CI, 12.72–41.34).

### Detection of HEV RNA in Human Samples

Following serology testing, samples from 16 patients with acute hepatitis were selected for HEV-RNA detection to confirm acute nature of HEV infection (Figure 1). HEV RNA was detected in 2 out of 16 samples. For one positive patient, HEV RNA was detected in the plasma sample, but not in feces. For the second positive patient, HEV RNA was detected only in the serum sample, the other types of samples not being available for analysis. The two HEV RNA positive patients belonged to the 41–60 age group and were hospitalized during 2016. In the case of the patient whose samples were obtained directly from the hospital, the attending physician anamnesis revealed the patient's habit of consuming pork meat bought from local store, but there were no details regarding the thermic treatment of the meat. The patient lived in the urban area and didn't own any domestic animal.

Thirteen HEV Ab positive serums from patients without liver disease were tested for HEV RNA using RT-nested PCR. All samples were negative for viral RNA.

### HEV Sequence Analysis

**Figure 2:**
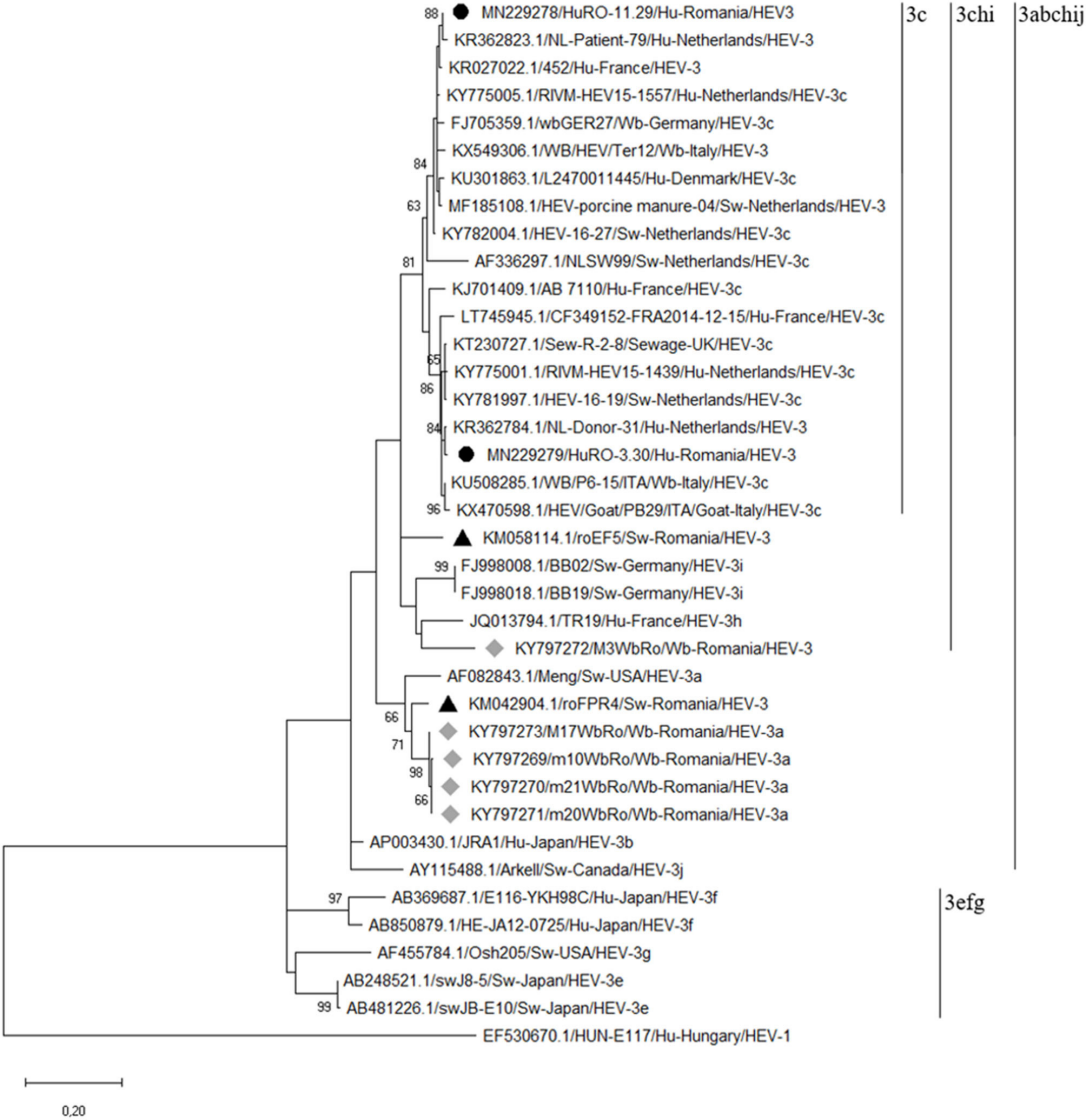
Phylogenetic tree based on the alignment of the generated sequences with the best hits to our sequences in GenBank and with the reference strains of each HEV3 subtype. The tree was constructed on the basis of a 348 bp fragment of the capsid protein-encoding region (ORF-2). The phylogenetic tree was built using the Maximum Likelihood method based on the Kimura 2-parameter model with 1,000 replicates. A human HEV-1 strain (EF530670.1/) was used as out group (the human HEV strains obtained in our study are highlighted by black bulleted; the gray diamonds indicated the wild boar HEV strains; the black triangles indicated the swine HEV strains previously characterized in eastern Romania).

The two RT-nested PCR products positive for HEV RNA were sequenced. Each sequence was manually edited and, by overlapping the complementary ones, a 333 nt sequence for the HuRO-3.30 isolate (GenBank: accession number MN229279) and a 332 nt sequence for HuRO-11.29 isolate (GenBank: accession number MN229278) were obtained. The bioinformatic analysis revealed that these isolates belong to HEV genotype 3. Based on the alignment of our sequences with the best hits in GenBank, the obtained isolates were assigned to the HEV-3c subtype (3chi monophyletic group) (Figure 2).

BLAST analysis showed that the percentages of nucleotide identity between our human HEV isolates and the homologous isolates from the GenBank database, range from 95 to 98%. The percentage of nucleotide identity between the two isolates is 92%. Moreover, BLAST analysis has shown that our isolates are related to both humans and animals HEV strains circulating in Europe.

The HuRO-3.30 isolate had a maximum 98% nucleotide identity with a Dutch human strain isolated in 2013 (GenBank accession no.KR362784). The maximum nucleotide identity (97%) of HuRO-3.30 isolate and GenBank HEV3c animal isolates was observed for an Italian wild boar strain (GenBank accession no. KU508285) and a swine isolate from the Netherlands (GenBank accession no. KY781997). Also, a 96% nucleotide identity was observed with a goat HEV strain from Italy (GenBank accession no. KX470598) and with two human strains isolated in Netherlands (GenBank accession nos. KY775030 and KY775001).

The HuRO-11.29 isolate had 98% maximum nucleotide identity with two Dutch human isolates (GenBank accession nos. KR362823 and vKY775005). This Romanian HEV isolate showed a 97% nucleotide identity with the manure HEV strain from Netherlands (GenBank accession no. MF185108) and a 96% with two wild boar isolates: one from Italy (GenBank access no. KX549306) and one from Germany (GenBank accession no.FJ705359). The latter isolate represents the reference sequence for subtype 3c (17).

The nucleotide identity between the human isolates of this study and those previously obtained from Romanian pig and wild boar range between 83 and 87%.

## Discussions

According to data published by the European organizations with a major role in ensuring health status - ECDC and EFSA, the infection with HEV is endemic in Europe, with an upward trend over the last 10 years (19). Previous serological studies performed in Romania have shown that human HEV infection is present in the Eastern region of the country (9, 20, 21).

The aim of the serologic study was to evaluate the presence of HEV antibodies in different patients' categories, with clinical signs of acute hepatitis and with no clinical signs. The main reasons of this choice were determined by the limited data regarding hepatitis E in Romania and the fact that in Europe, most of the infections are asymptomatic.The results of HEV total antibodies detection in patients with acute hepatitis of unknown etiology revealed that HEV infection could have been the cause of liver disease in 29.2% (14/48) of the analyzed patients. Subsequently, the serological screening of HEV IgM antibodies in some of these patients (44/48) and those with non-A, non-B acute hepatitis (*n* = 46), point out that 10% (9/90) of the analyzed subjects had an acute HEV infection at the time of hospitalization.

In the context of this study, analysis and interpretation of HEV testing results on patients with hepatitis of unknown etiology (negatives for HAV and HBV infections) highlights the need to implement a national program for the prevention, surveillance and control of hepatitis E infections in Romania. The lack of such a program makes HEV infection underdiagnosed and makes it difficult for medical staff to determine the cause of the liver disease. Moreover, the ECDC report from September 2019, states the establishment of effective surveillance systems for hepatitis E in EU countries.

Our previous results on the detection of HEV IgG antibodies in patients with hepatitis B or C from Eastern Romania, revealed that 12% (3/25) of the individuals, had been exposed to HEV (20). Allover, the results of our 2 investigations highlights that HEV infection is frequent and possibly coexists with other viral hepatitis. The results of the present study show also that three patients with TB had been exposed to HEV. It is not known if HEV exposure happened before or after TB infection and was associated or not to acute hepatitis.

The serological results of investigations in women patients in postnatal confinement revealed 10 HEV seropositive patients out of 37. In developing countries where HEV-1 is endemic, HEV infection in pregnant women can lead to a high mortality rate (21%), especially if this occurs in the third trimester of pregnancy (22). In Europe, the most commonly reported genotype is HEV-3, and up to now, no infection in pregnant women was associated with severe disease (23, 24). The lack of HEV IgM antibodies testing and the limitation of a single type of sample (serum) used for HEV RNA detection, did not allow us to draw a definite conclusion. However, the relatively low prevalence of HEV IgM antibodies or the lack of HEV RNA detection in pregnant women seems to be common reported in several European studies (25, 26). Also, a more recent study conducted by Renou et al. (27) suggest that HEV infection is a sporadic event during pregnancy even in European regions with high seroprevalence rates.

In Europe, the reported seroprevalence rates vary from one country to another and within the same country from one study to another. The meta-analysis presented by Hartl et al. (28), pointed out that these differences were due to the used test, to the geographic region and the analyzed group, these factors limit the comparison of seroprevalence rates between studies. However, the existing data analysis has revealed a higher seroprevalence rate in individuals exposed to animals and that it increases with age (29). Our results are consistent with data published in the literature, the highest seroprevalence being recorded in people older than 60 years.

Hepatitis E seroprevalence rates in both patient categories: patients with acute hepatitis and patients without liver disease did not vary considerably (29.2 vs. 32.5%, *p* = 0.3). Related to recent European reports, some data have pointed out that blood donors HEV seroprevalence rate is higher than the one reported in patients with acute hepatitis (30). In France, it was suggested that the discrepancy between the seroprevalence rates observed between these two categories could be associated with the high number of asymptomatic cases of HEV infection (70%) (31). In our study we did not notice differences between HEV seropositivity depending on the living condition of the patients- rural and urban area, these results being in line with those reported in the literature (32).

Investigations regarding HEV seroprevalence in medical staff from ECHSPF led to no positive sample for HEV Ab. Results of a previous study conducted by Voiculescu et al. (11), in human population from Southern and South-Eastern Romania showed a 13.98% (13/98) seroprevalence of HEV IgG antibodies in surveyed medical staff. It is known that the primary route of transmission of HEV infection is fecal–oral, the secondary transmission by direct contact with an infected person not being reported, not even in the context of an outbreak (33).

The molecular investigations revealed two patients positive for HEV RNA out of nine patients HEV IgM positive. Regarding pattern of viremia and fecal shedding in HEV infection, similar results were described by Goel et al. (34). The overlap between the serological results and the molecular analysis demonstrate a recent infection at the time of hospitalization. The HEV RNA positive sample from the hospitalized ECHSPF patient, allowed us to collect data on potential risk factors associated to HEV infection. The patient had consumed pork meat from a local grocery store, which raises concern about the potential for transmission through consumption of contaminated food. In Europe symptomatic cases of hepatitis E are associated with consumption of pork meat and pork products. However, the number of cases directly related to the consumption of contaminated food remains limited, zoonotic transmission being indicated by genetic relatedness between human viral strains and pig, wild boar and deer viral isolates, or with those isolated from meat products, available in GenBank (8).

Genetic characterization of the two Romanian human HEV strains revealed that they belong to the genotype 3, subtype 3c. This is the most common subtype identified in Europe, in the recent years the number of reports having an upward trend in countries like the Netherlands, England, France, Spain and Italy. The phylogenetic analysis based on a 348 nt fragment of the capsid protein-encoding region (ORF-2), has revealed that these two isolates are genetically related to human, pig and wild boar HEV strains in the Netherlands, Italy and Germany, but also with the isolates from pigs' manure in the Netherlands or from sewage in England. Furthermore, the phylogenetic analysis revealed that the highest nucleotide identity was observed toward the isolates from humans and swine in the Netherlands. In order to replace predominant subtypes, HEV-3e, f, g, with the HEV-3c subtype in humans in England, it has been suggested an association with pig meat or pork products imports from other EU countries where this subtype is prevalent, like the Netherlands. The imports of pork grew considerably in Romania, 83.4 thousand tons in the first half of 2016, in the same period of the previous year the imported quantity being of 74.6 thousand tons. Germany, Spain, Hungary, the Netherlands, Italy, Denmark, France are among the main supplier countries in Europe and Romania. Following these findings, we can suspect a possible association of the detected infections with the consumption of meat or products derived from imported pork. If this presumption is true, we can discuss of an endemic infection with HEV-3c in Europe.

Our previous studies have shown that different HEV-3 subtypes are circulating in pigs and wild boars in the Eastern part of the country, and that the isolated strains are related to those isolated from autochthonous cases of HEV infection in humans in Europe (10). The phylogenetic analysis showed that the strains isolated in this study are included in the “3chi” monophyletic group together with the strains previously identified in wild boars and pigs. The wild boar strains included in the HEV-3a subgroup have been shown to be genetically related to a strain isolated from swine (9, 10). In some studies, more than two HEV subtypes of the analyzed species were identified. For example, in Germany and Italy, various subtypes were identified in the analyzed wild boars, in a single study (33, 35). In England, the isolated human HEV-3 strains were grouped into two different clusters, 1 (subtypes 3e and 3f) and 2 (subtypes 3c, 3h, and 3i), the authors of the study demonstrating the presence of autochthonous infections (36). Based on these remarks, we can assume that different HEV subtypes are circulating in the human population and animals, in Eastern Romania.

In conclusion, in order to confirm these assumptions, future studies including a large number of samples should provide a direct evidence of HEV zoonotic infection by including research on all elements involved in food production, from the water sources used in animal husbandry to the final product consumed by the population. In addition, phylogenetic analysis of sequences from different regions of the HEV genome could be useful in studying the deep phylogenetic relationships between strains with distinct origins.

## Data Availability Statement

The datasets presented in this study can be found in online repositories. The names of the repository/repositories and accession number(s) can be found below: https://www.ncbi.nlm.nih.gov/genbank/, MN229278; https://www.ncbi.nlm.nih.gov/genbank/, MN229279.

## Ethics Statement

The studies involving human participants were reviewed and approved by Sfanta Parascheva Infectious Diseases Hospital of Iasi. Written informed consent to participate in this study was provided by the participants' legal guardian/next of kin.

## Author Contributions

DP, AA, and GS contributed to conception and design of the study. DP, LC, IR, AC, CR, VG, AA, AV, and DT organized data collection. DP, CR, DA, LO, LC, VG, and NP contributed to data interpretation and statistical analysis. DP, AA, GS, and NP wrote sections of the manuscript. All authors contributed to manuscript revision, read, and approved the submitted version.

## Conflict of Interest

The authors declare that the research was conducted in the absence of any commercial or financial relationships that could be construed as a potential conflict of interest.
